# Efficient Generation of Spectrum-Manipulated Few-Cycle Laser Pulses through Cascaded Dual-Chirped OPA

**DOI:** 10.3390/ijms22136887

**Published:** 2021-06-26

**Authors:** Zuofei Hong, Han Zhang, Shaolin Ke

**Affiliations:** Hubei Key Laboratory of Optical Information and Pattern Recognition, Wuhan Institute of Technology, Wuhan 430205, China; 22010010032@stu.wit.edu.cn

**Keywords:** ultrafast light source, optical parametric amplification, few-cycle laser pulses

## Abstract

The cascaded dual-chirped optical parametric amplification (DC-OPA) is presented for efficient generation of few-cycle infrared (IR) laser pulses. The input pulses are strategically chirped to optimize the phase-matching bandwidth in each nonlinear crystal, and four regions of the signal spectrum are amplified in cascaded crystals with different cutting angles, enabling flexible manipulation of the output spectrum. Broadband gain and high conversion efficiency are simultaneously achieved owing to the cascaded-crystal arrangement, the signal pulse duration of 4.2 cycles is obtained with 11.7-mJ pulse energy, corresponding to a conversion efficiency of 39.0%. The proposed scheme offers a robust and simple approach to pushing the phase-matching bandwidth limits introduced by the nonlinear crystal, which manifests great prospect in various researches involving ultrafast optics and strong-field physics.

## 1. Introduction

During the past decades, femtosecond (fs) laser with the pulse duration down to few optical cycles has become a workhorse in a wide range of applications, the superb temporal resolution accompanied with ultrahigh peak intensity provides unprecedented condition in numerous laser-matter interaction researches such as time-resolved spectroscopy, strong-field physics, fs-laser direct writing, and nonlinear optics [1,2,3,4,5,6,7]. Owing to the intriguing properties including ultrabroad bandwidth, high single-pass gain, and broadband tunability, optical parametric amplification (OPA) and optical parametric chirped pulse amplification (OPCPA) have been widely employed to down-convert the commercially available fs laser for generating ultrashort pulses in the near-infrared (NIR) to mid-infrared (mid-IR) regions [8,9,10,11].

In order to achieve ultrabroad spectral bandwidth and produce few-cycle pulses in OPA, multiple efforts have been made to overcome the restriction on the phase-matching (PM) bandwidth of a nonlinear crystal. The parametric waveform synthesizer (PWS) has proven to be an effective approach to coherently synthesize multiple pulses with stabilized carrier envelope phase (CEP) from different OPAs [12,13]. Rather than amplification in the temporal domain, the frequency-domain OPA (FOPA) presents an innovative design to amplify slices of the broadband signal individually in different crystals by projecting the amplified spectrum onto the Fourier plane [14,15]. Despite the capability of combining several spectral regions for an outstanding output bandwidth, the implementation of either PWS or FOPA involves splitting and recombining the signal spectrum, which requires high stability and precision in the handling of interacting pulses.

The multiple-crystal OPA scheme is proposed as a simpler alternative [16]. Different spectral ranges are successively amplified in collinearly arranged nonlinear crystals, and a synthesized spectrum that supports few-cycle output pulses is obtained as a result. Unfortunately, due to the ultrashort duration of the employed pulses in multiple-crystal OPA, the output spectrum is extremely sensitive to their group delay. On the other hand, limited by the damage threshold of the nonlinear crystal, further enhancing the pulse energy using more powerful Ti:sapphire laser faces technical difficulties.

One of the most promising methods for pulse energy scaling is the DC-OPA scheme, in which the input fs pulses are linearly stretched to picosecond (ps) level before entering the nonlinear crystal [17,18,19]. Chirping the interacting pulses enhances the energy capacity of the system within the restriction of the crystal damage threshold, and opens up the possibility of employing multimillijoule fs laser as the OPA pump.

In this paper, we present a novel cascaded DC-OPA scheme for efficient generation of few-cycle IR pulses via spectrum manipulation. The input pulses are both chirped to not only minimize phase mismatch for all signal wavelengths, but also decrease the peak pump intensity, which therefore enables scaling the signal energy by using more energetic pump pulses. Consecutive regions of the signal spectrum are amplified in cascaded nonlinear crystals with different cutting angles, intensities of each spectral region can be flexibly adjusted by selecting appropriate crystal lengths. As a consequence, the synthesized ultrabroad bandwidth that corresponds to a sub-four-cycle pulse duration is obtained. Moreover, favorable conversion efficiency is achieved owing to the total crystal thickness, and multimillijoule pulses with even shorter durations are potentially available by further optimizing the input pulse parameters.

## 2. Method

Firstly, the PM condition in a typical DC-OPA is illustrated. In a nonlinear crystal with a certain cutting angle, the pump wavelength is phase-matched to a limited signal spectrum range. The corresponding relationship between phase-matched pump and signal wavelengths is essential for the gain bandwidth in OPA, especially when the involved pulses are chirped. For both the pump and seed pulses in DC-OPA, different frequency components appear at different time delays, the synchronization condition between their instantaneous wavelengths follow a pattern defined by the dispersions of each pulse. When this pattern matches with the PM corresponding relationship, efficient amplification can be realized for all spectral components of the signal pulse, thus realizing broadband gain. As presented in previous works, such a feature can be applied in a chirp-compensation scheme and ultrashort laser pulses have been obtained [20,21,22].

**Figure 1:**
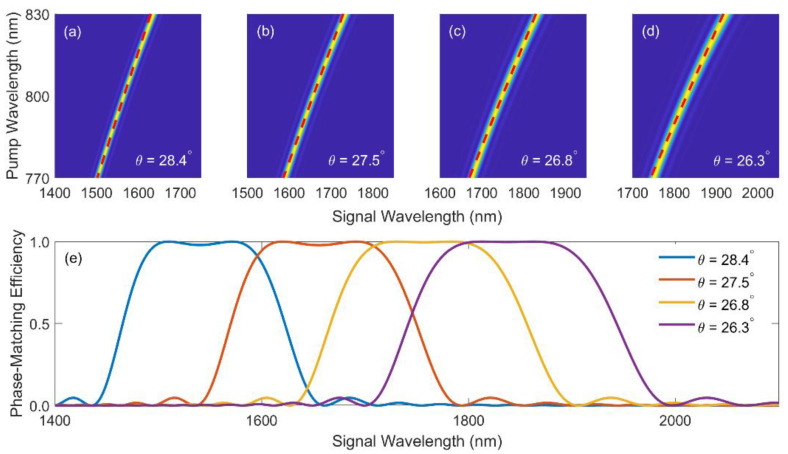
Phase-matching efficiency in type-II BBO crystals cut at θ = 28.4° (**a**); 27.5° (**b**); 26.8° (**c**), and 26.3° (**d**), respectively. The red dashed lines depict the linear fit of the phase-matching curve; (**e**) Signal phase-matching efficiency in a linear-fitting dual-chirped geometry.

As an example, we consider the cases in 2 mm beta barium borate (BBO) crystals with different cutting angles and calculate the PM efficiency sinc^2^(ΔkL/2) for varying pump and signal wavelengths, as shown in Figure 1a–d. The crystals are cut for type-II phase-matching with θ = 28.4° (a), 27.5° (b), 26.8° (c), and 26.3° (d), respectively. The pump wavelength is between 770 nm and 830 nm, which is the center part of a Ti:sapphire fs laser spectrum. It is observed in each figure that the phase-matched signal wavelength varies with the pump wavelength. The most efficient part of each figure can be fit linearly, as denoted by the red dashed lines, which suggests the possibility of employing linearly chirped pulses in the chirp-compensation scheme. Note that the linear chirp indicates linear relationship between temporal delay and instantaneous frequency, whereas Figure 1a–d are plotted with respect to the wavelengths, which explains the slight curve of the “linear-fit” dashed lines.

When the input pulses are chirped according to the red dashed lines, the resulted PM efficiency is plotted in Figure 1e. For all crystal angles, trapezoid-shaped curves are observed with the efficiency maximized around the central wavelengths and slowly decrease at the edges. Despite the desirable PM condition, the gain bandwidths are at most 200 nm, which are insufficient for few-cycle pulse durations. In order to exploit this feature while realizing an ultrabroadband output spectrum, the novel DC-OPA scheme combining these narrowband spectra with cascaded crystals is therefore proposed.

**Figure 2:**
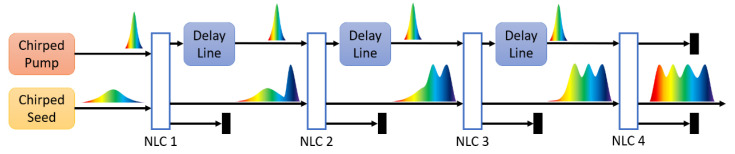
Schematic setup of the cascaded DC-OPA. NLC—nonlinear crystal.

The conceptual layout of the cascaded DC-OPA is depicted in Figure 2. A narrowband pump and a broadband seed are both stretched with negative group delay dispersions (GDDs), with the chirp ratio determined by the gradient of the red dashed lines in Figure 1a–d. Due to their bandwidth difference, the pump pulse is evidently shorter than the seed pulse, hence only part of the signal spectrum can be overlapped and amplified within the pump profile.

In the first nonlinear crystal (NLC), the pump pulse overlaps with the leading edge of the seed, only the high-frequency part of the signal gets amplified. Afterwards, the pump pulse is delayed with a delay line (DL) and synchronized to the signal spectrum with slightly lower frequency, whereas the idler pulse is removed to inhibit upconversion in the following NLC. Similar procedures are repeated before the third and fourth NLCs, and the pump pulse is synchronized with the trailing edge of the seed in the final NLC, amplifying the low-frequency part of the signal. Four spectral regions are successively amplified in the cascaded crystals, each with optimized thickness, cutting angle and pulse delay, respectively. Consequently, ultrabroadband signal combining the phase-matched spectral regions is obtained.

## 3. Results

In this article, the parametric amplification process is simulated by numerically solving the coupled-wave equations. Details on the numerical model can be found in [17]. The effects of nonlinear interaction, group velocity mismatch, and dispersions up to the fourth order have been considered in the model, which is sufficient to reveal the basic principles of the proposed scheme.

Cascaded DC-OPA using type-II BBO crystals with the angles of θ = 28.4°, 27.5°, 26.8°, and 26.3° are investigated. The system is pumped by a Gaussian-shaped 30-fs, 30-mJ, 800-nm Ti:sapphire laser pulse, and the seed is centered at 1700 nm with a transform-limited (TL) pulse duration of 15 fs and pulse energy of 100 μJ. Both the pump and seed beams are Gaussian-shaped with a diameter of 10 mm in the spatial domain. The pump and seed pulses are linearly stretched to 1.66 ps and 6.65 ps with negative GDDs of −20,000 fs^2^ and −36,000 fs^2^, respectively. The resulted pump-seed chirp ratio is 1.8, which coincides with the gradient of red dashed lines in Figure 1a–d. The temporal delays between the pump and signal pulses in four crystals are −3440 fs, −1000 fs, 1120 fs, and 2820 fs, respectively. Negative delay value indicates that the pump pulse arrives at the crystal before the seed pulse, overlapping with its high-frequency components, and vice versa. Ideal PM condition can be expected using the optimized combination of cutting angles, chirp ratio, and the pulse delays.

The crystal thicknesses are selected with the objective of obtaining largest signal bandwidth while maximizing the conversion efficiency. Under this consideration, we employ four crystals of 2.7 mm, 2.8 mm, 3.1 mm, and 4.2 mm, respectively. The peak intensity of the pump pulse is highest in the first crystal; thus the shortest crystal length is used. As the pump energy depletes, longer crystals are required in the later stages to ensure similar output spectral intensity.

**Figure 3:**
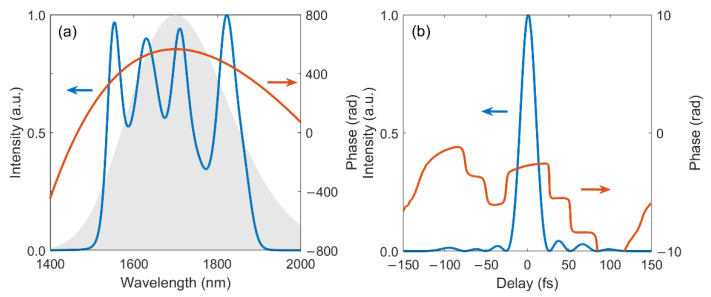
(**a**) Spectral intensity (blue) and phase (red) of the output signal pulse, the input seed spectrum is shown as the gray area; (**b**) Temporal intensity (blue) and phase (red) of the linearly compressed signal pulse.

The spectral-temporal characteristics of the output signal pulse obtained with the abovementioned parameters are plotted in Figure 3. The gain efficiency in OPA is determined by both the PM condition and the pump intensity. Given the excellent PM efficiency within the pump profile, it can be inferred that the shape of each peak in the spectrum is mainly inherited from the pump intensity distribution. As a result, a four-peak signal spectrum is achieved, in which all peaks are near Gaussian-shaped and each one corresponds to amplification in each crystal.

The full-width-at-half-maximum (FWHM) bandwidth of the spectrum is 324 nm, which is broader than the 285 nm FWHM bandwidth of the seed and supports a TL pulse duration of 20.4 fs, corresponding to 3.6 optical cycles. A quadratic spectral phase (red line) is observed in Figure 3a, suggesting a linear temporal chirp with good compressibility. The temporal profile of linearly compressed signal pulse is plotted in Figure 3b with a near-TL 3.8-cycle pulse duration of 21.4 fs; effective compression is revealed by the flat temporal phase within the main pulse.

**Figure 4:**
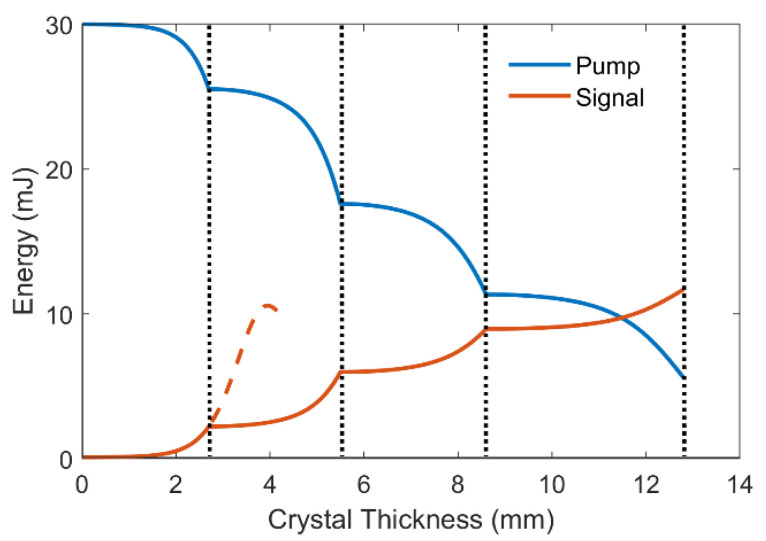
Pump (blue) and signal (red) energy evolution with respect to the total crystal length, the red dashed line denotes signal energy evolution without the cascaded-crystal geometry, and the dotted lines indicate interfaces between adjacent crystals.

The utilization of cascaded crystals not only broadens the gain bandwidth in the DC-OPA system, but also provides sufficient crystal length for high energy conversion. As plotted in Figure 4, the pump energy is consistently being transferred to the signal pulse during the amplification in four crystals, which leads to the energy depletion of over 80%. After the total crystal length of 12.8 mm, the signal energy of 11.7 mJ is achieved with the conversion efficiency of 39.0%, which is close to the 47% quantum efficiency of the parametric process.

To elaborate the mechanism of the cascaded amplification, we define the temporal regions of the signal pulse that becomes amplified in first to fourth crystals as R1, R2, R3, and R4, respectively. In a negatively chirped signal pulse, R1 contains the highest-frequency components and R4 contains the lowest-frequency components. After the first crystal, the intensity of R1 is increased while the pump intensity is reduced. Further amplification in the same crystal will result in the saturation of R1 (red dashed line in Figure 4) as well as the gain-narrowing effect originated from the relatively shorter pump pulse. Therefore, the pump is delayed and synchronized to R2 instead. It is noteworthy that as the pump delay is adjusted, the signal and idler intensities in R1 are higher than the pump, which would lead to back-conversion and the declination of signal energy. The idler pulse is hence removed to circumvent such detrimental effect [23,24]. In this case, low-intensity R2 is amplified in the second crystal, yet the high-intensity R1 is not depleted due to back-conversion. The process is repeated in the following crystals, and the signal pulse gets continuously amplified in all crystals, leading to the excellent conversion efficiency.

The adjustment of pump-signal delay and removal of idler pulse ensure that the amplified signal components are strictly limited within the pump pulse envelope. For instance, when R3 is amplified in the third crystal, the intensities of R1, R2, and R4 remain unchanged. Such setup allows individual manipulation of each temporal/spectral region of the chirped signal pulse by selecting appropriate crystal length, which could benefit OPA systems with various laser conditions.

**Figure 5:**
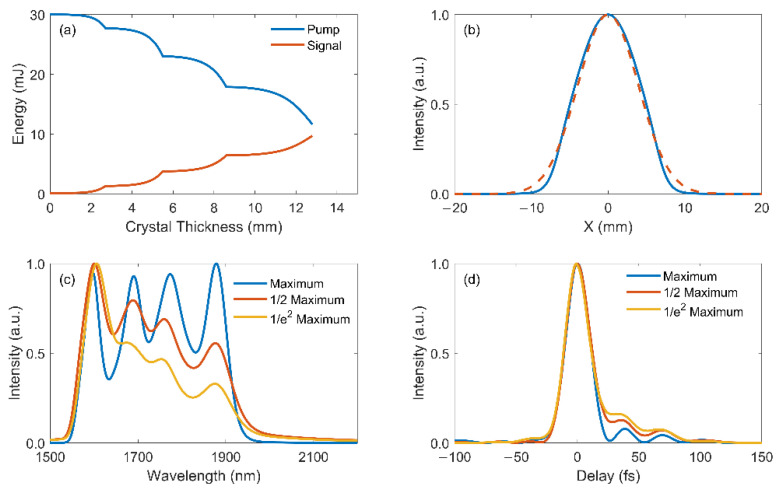
Results in 2+1D simulation: (**a**) Pump (blue) and signal (red) energy evolution with respect to the total crystal length; (**b**) Spatial profile of the linearly compressed signal pulse, red dashed line is the Gaussian fit. Spectra (**c**) and temporal profiles (**d**) of the linearly compressed signal pulse at maximum intensity (blue), half maximum intensity (red) and 1/e^2^ maximum intensity (yellow) positions in the beam.

In addition to the spectral and temporal properties, the laser beam is produced with a Gaussian distribution in the spatial domain with varying intensity transversely, which also needs to be taken into account. The simulation including spatial effects is carried out in a 2+1 dimensional (2+1D) numerical model, where the spatial profile and diffraction of interacting beams are added to the above investigations. All the parameters of the pump and seed pulses are kept unchanged, and the results are depicted in Figure 5.

The above output parameters are achieved with the peak intensity of the pump beam whereas the beam edge is weaker than the center, leading to a decrease in the total efficiency. As observed in Figure 5a, the signal pulse of 9.7 mJ is obtained with a slightly lower conversion efficiency of 32.3% compared to the one-dimensional (1D) case, which still suggests sufficient conversion in the cascaded crystals with the long total thickness. The compressed signal beam shows a smooth spatial profile in Figure 5b that can be fitted with a Gaussian curve (red dashed line), and the good beam quality is preserved after cascaded amplification.

The spectral-temporal characteristics across the beam is investigated and depicted in Figure 5c,d. The spectra and temporal profile of the compressed signal pulse at the half maximum intensity (red lines) and 1/e^2^ maximum intensity (yellow lines) are plotted in comparison to the peak intensity point (blue lines) in the beam. Distorted spectrum is observed near the beam edge, which is resulted from the decreased gain efficiency in later stages due to the lower pump intensity. Even though the signal bandwidth is narrower with spectral distortion, the pulse is still compressible to few-cycle durations with a nearly identical main pulse as the beam center, and only weak side lobes are observed post pulse. The system manifests excellent validity with its well-preserved beam quality and high spatial-temporal uniformity in the 2+1D simulations.

## 4. Discussion

Following the above illustration, we investigate the performance of cascaded DC-OPA systems with narrower seed bandwidth. Seed pulses with TL durations varying from 15 fs to 25 fs are selected with fixed pulse energy, center wavelength, and chirp, and the employed pump parameters are the same as above. By changing the seed bandwidth, the stretched pulse has a different pulse duration, and the intensities of unamplified R1–R4 are different from the previous case, meanwhile the fixed GDD implies the constant pump-seed chirp ratio. Therefore, the crystal angles are still 28.4°, 27.5°, 26.8°, and 26.3° whereas the crystal lengths have to be updated in every case accordingly. The optimized parameters and subsequent results are summarized in Table 1.

The seed pulse with a longer TL duration contains a narrower spectrum, after being stretched with the same GDD, the edges (R1 and R4) of the chirped seed pulse become weaker whereas the center (R2 and R3) are more intense. In order to generate a signal spectrum with four peaks of similar intensities, the lengths of first and fourth crystals should be longer and the other two crystals should be shorter. Consequently, the crystal lengths listed in Table 1 are selected. With longer crystals amplifying R1 and R4, the gain-narrowing effect becomes more influential on the edges of the signal spectrum, resulting in a slightly narrower output bandwidth. Nevertheless, the spectrum manipulation with cascaded crystals still significantly broadens the signal bandwidth compared to the seed. In the meantime, the effect of seed bandwidth on the conversion efficiency is negligible owing to the sufficient total crystal thickness.

**Figure 6:**
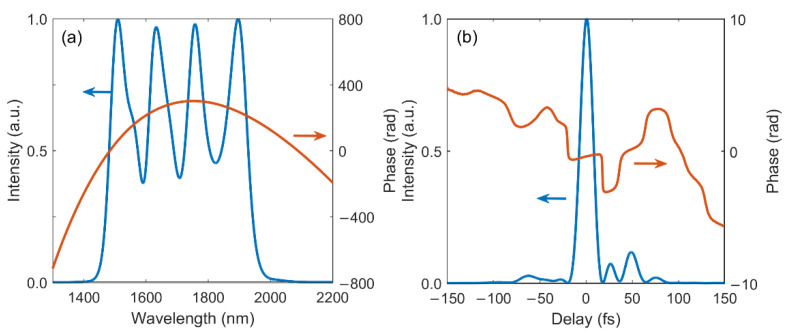
(**a**) Spectral and (**b**) temporal characteristics of the output signal pulse generated from a broadband-pumped cascaded DC-OPA system.

As discussed above, the PM bandwidth in DC-OPA is restricted by the bandwidth of the pump pulse, it is therefore natural to infer that a broadband pump could benefit the gain bandwidth in the proposed scheme [25,26]. The performance of cascaded DC-OPA system pumped by a pump pulse with a shorter TL duration of 18 fs is investigated. The pump pulse is stretched with the same GDD to maintain the pump-seed chirp ratio, a longer pulse duration after chirping is obtained compared to the narrowband pump condition. With the longer stretched pump pulse, it can overlap with a larger range of the stretched signal pulse (i.e., phase-matching with a broader signal bandwidth) during amplification. As a result, the cutting angles of the cascaded BBO crystals are tuned to 29.0°, 27.5°, 26.3°, and 25.7°; the pump-seed delays in four stages are optimized to −4900 fs, −1035 fs, 2810 fs, and 5135 fs as well, and the crystal thicknesses are adjusted to 3.6 mm, 3.3 mm, 3.8 mm, and 3.3 mm, respectively. The spectral-temporal performance of the broadband-pumped system is shown in Figure 6.

The shape of the output signal spectrum with four near-Gaussian peaks is maintained, the bandwidth of each peak is broadened compared to narrowband pump case. The combination of the four broader spectra leads to a broader signal FWHM bandwidth of 448 nm, which supports a 2.7-cycle TL duration of 15.5 fs. The changes in pump bandwidth and duration barely affect the spectral phase of the signal, a quadratic phase curve is again observed. The temporal profile of linearly compressed signal pulse is plotted in Figure 5b, efficient compression is achieved as indicated by the flat temporal phase (red curve), resulting in a 2.9-cycle pulse duration of 16.3 fs.

Aside from the ultrabroad gain bandwidth, the prospect of scaling pulse energy is also of interest. An intriguing property of DC-OPA is the capability of pumping OPA using ultrahigh pulse energy, the peak intensity of the pump pulse is limited under the damage threshold of the nonlinear crystal through linear chirp. Same feature applies to the proposed scheme, the potential of stretching input pulses with larger GDDs enables using higher energy as the pump. In addition, the peak intensity of the stretched pump pulse in the above condition is only 10.3 GW/cm^2^, which is far from the damage threshold of BBO crystal. By increasing the pump intensity, broader gain bandwidth with shorter crystals can be expected, which could further improve the performance of cascaded DC-OPA system.

The above investigations consider ideal conditions where the parameters are optimized for the best output characteristics. However, experiments contain errors and instabilities that affect the system performance. In order to determine the influence of such practical issues, we have simulated the cascaded amplification process with random errors introduced to the pump-seed delay, nonlinear crystal angles and lengths. The simulation is repeated 200 times, each time with a random error in all four stages, and all other parameters remain the same as in the above section. The ranges of the errors are limited within ±50 fs, ±0.05°, and ±0.1 mm for the delay, angle and length, respectively, and the obtained results are listed in Table 2.

As shown in the table, despite the slight deterioration and instability due to the errors, the RMS value of the FWHM bandwidth, compressed pulse duration and conversion efficiency is barely changed compared to the previous results, and high stability is achieved with only ~1% RMS fluctuations. The strict requirement on the pulse delay and the crystal parameters are largely relaxed by the ps-level durations of the chirped pulses and the long total crystal lengths. Therefore, the proposed scheme is an appropriate approach for experimentalists working in the ultrafast-laser-related research fields.

Other than the NIR region discussed above, the spectra-combining nature of the cascaded DC-OPA indicates its potential application in other wavelengths. As an example, we have investigated the scheme using LiIO_3_ crystals, which are more suitable for mid-IR pulse generation owing to its higher transmission in the spectral region compared to BBO crystals. With the dispersion property of the LiIO_3_ crystal, the group velocity mismatch (GVM) between signal and idler can be eliminated near 1 μm, enabling broadband pulse generation at 3.5 μm [9]. However, generating few-cycle mid-IR pulses centered at a shorter wavelength (<3.5 μm) is technically difficult with such crystal.

**Figure 7:**
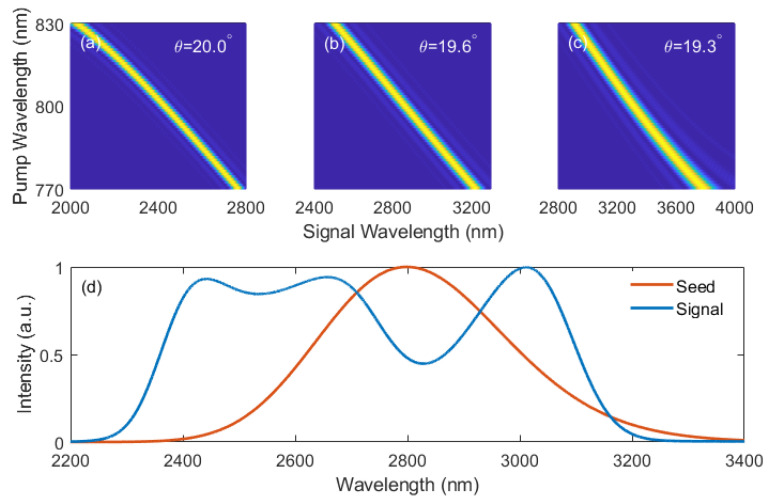
Phase-matching efficiency in type-I LiIO_3_ crystals cut at *θ* = 20.0° (**a**); 19.6°(**b**), and 19.3° (**c**), respectively; (**d**) Output signal (blue) and input seed (red) spectra in the cascaded DC-OPA based on LiIO3 crystals.

The phase-matching efficiencies of 800 nm pump and mid-IR seed in LiIO_3_ crystals with the cutting angles of 20.0°, 19.6°, and 19.3° are calculated and presented in Figure 7a–c, respectively. The phase-matched pump and seed wavelengths clearly show a linear trend with similar pump-signal chirp ratio, revealing the possibility of applying the cascaded DC-OPA scheme in the crystals. The process is simulated with the same pump pulse as above and a 2.8 μm, 30 fs, 100 μJ pulse as the seed. The GDDs of the input pulses are optimized to 20,000 fs^2^ and −20,000 fs^2^ for the pump and seed pulses, respectively. The pump-seed chirp ratio of −1 is used to better fit the phase-matching curve.

The crystal lengths of 2.3 mm, 1.7 mm, and 3.2 mm are selected under the same principle of generating three spectrum peaks with similar intensities, and the spectrum (blue line) in Figure 7d is achieved. The mid-IR spectrum with a FWHM bandwidth of 744 nm is achieved, which is obviously broader than the seed spectrum (red line in Figure 7d). Like the BBO-based system, the signal spectrum supports simple linear compression down to 23.2 fs, corresponding to 2.5 optical cycles; meanwhile, the conversion efficiency of 19.3% is realized simultaneously. Based on the above illustrations, it is reasonable to infer that the proposed scheme is of great potential in generating few-cycle laser pulses centered at various spectral regions with optimized crystal parameters.

## 5. Conclusions

In conclusion, we propose and numerically investigate the cascaded DC-OPA scheme for efficiently generating few-cycle laser pulses. Pump and seed pulses with strategically tailored linear chirps interact in cascaded nonlinear crystals, whose cutting angles are optimized for broadband amplification of different spectral regions. Ultrabroadband gain is realized by manipulating the spectrum with carefully selected crystal lengths, few-cycle pulses with excellent conversion efficiency and compressibility are obtained. Performance of the scheme can be potentially improved with more favorable pump conditions. Moreover, the system is applicable in various wavelength regions by employing more appropriate nonlinear crystals such as LiIO_3_, KNbO_3_, etc. Coherent synthesis of multiple spectra is realized in a simple collinear geometry, in the absence of demanding experimental conditions, which is beneficial to the development of applications related to ultrafast and ultra-intense laser pulses.

## Figures and Tables

**Table 1:** Performance comparison between cascaded DC-OPA with various TL durations of the seed pulse.

Seed		Crystal Length (mm)				Signal		Conversion Efficiency (%)
TL Duration (fs)	Bandwidth (nm)	1	2	3	4	Bandwidth (nm)	TL Duration (fs)	
15.0	285	2.7	2.8	3.1	4.2	324	20.4	39.0
17.5	244	2.7	2.7	3.0	4.2	318	21.0	38.4
20.0	214	2.8	2.7	3.0	4.4	316	21.3	38.5
22.5	189	2.9	2.6	2.9	4.4	308	21.8	37.0
25.0	171	3.0	2.6	2.9	4.6	307	22.0	37.4

**Table 2:** Fluctuations of system performance with error.

Error Type		Pump-Seed Delay	Crystal Angle	Crystal Length
Error Range		±50 fs	±0.05°	±0.1 mm
Root-Mean-Square (RMS) Value	FWHM Bandwidth	213.9 THz	212.7 THz	210.6 THz
	Compressed Pulse Duration	21.3 fs	21.3 fs	21.4 fs
	Conversion Efficiency	38.9%	38.8%	38.9%
Fluctuation	FWHM Bandwidth	0.42%	0.83%	1.25%
	Compressed Pulse Duration	0.36%	1.01%	1.20%
	Conversion Efficiency	0.57%	0.98%	1.00%
